# Potential of Carob Pulp Powder: Influence of Cultivar on Nutritional Composition, Antioxidant Activity, and Functional Properties

**DOI:** 10.3390/molecules30132715

**Published:** 2025-06-24

**Authors:** Carme Garau, Mónica Umaña, Miquel Llompart, Ismael Velázquez, Isabel Gálvez, Susana Simal

**Affiliations:** 1 Institute for Agri-Food and Fishing Research and Training of the Balearic Island, c/Eusebi Estada, 145, 07009 Palma de Majorca, Spain; mcgarau@irfap.es (C.G.); miquel.llompart@uib.cat (M.L.); ismael.mateu@gmail.com (I.V.); isagal.85@gmail.com (I.G.); 2 Department of Chemistry, Universitat de les Illes Balears, Ctra. Valldemossa Km 7.5, 07122 Palma de Majorca, Spain; monica.umana@uib.es

**Keywords:** carob pulp, by-product, antioxidant activity, nutritional composition

## Abstract

Carob pulp powder (CPP), a by-product of *Ceratonia siliqua* L., is rich in bioactive compounds with potential for functional foods. This study evaluated how genetic variability among cultivars affects the nutritional composition and functional quality of CPP. Nineteen cultivars from Majorca (13), Ibiza (4), and two open-flowering selections were grown under identical conditions in Majorca, Spain. CPP samples showed high dietary fibre (22.6–47.4 g/100 g dry matter), total sugars (22.5–62.5 g/100 g dm), and antioxidant activity (11.1–78.4 µmol TE/g dm, CUPRAC method). Significant differences among cultivars were observed in protein and fructose content, fatty acid profiles, antioxidant activity, colour, acidity, and functional properties like water- and oil-holding capacities. Principal component analysis distinguished the Ibiza cultivars by higher soluble solids, fructose, and stearic acid content but lower antioxidant activity. Open-flowering selections exhibited the highest antioxidant and water retention capacities. These results confirm that genetic origin strongly influences CPP’s nutritional and functional traits, endorsing its use as a natural, health-promoting ingredient for functional food development.

## 1. Introduction

The carob tree (*Ceratonia siliqua* L.) is a traditional tree that is grown natively in regions of the Mediterranean basin [1,2], on poor calcareous soils [3]. Due to its hardiness and drought resistance, it is well-suited to the Mediterranean climate and requires minimal maintenance for cultivation [4,5].

The carob pod is divided into two parts, pulp and seeds (90% and 10%, respectively) [6]. The pods vary in morphological characteristics, such as dimension, size, weight, shape, density, colour, and seed-pulp ratio, due to the difference between cultivars and climatic conditions [7,8]. Carob seeds, also known as carob beans, are distinguished by their hardness and are composed of 30–33% shell or cuticle, 42–46% endosperm or gum and 23–25% germ [5,9]. Due to their composition, carob beans have served as a valuable source of gum, with carob gum being a recognised thickening agent in the food industry (designated as E410). This gum boasts a significant galactomannan content, comprising 16–20% D-galactose and 80–84% D-mannose [9,10]. Carob beans hold significant importance within the food industry due to their physicochemical components with functional and flavouring properties and their nutritional benefits [6,11]. It is expected that in the next few years, the importance of the use of carob beans will increase worldwide due to the trend of discovering new natural and healthy supplements and the need for organic products, together with the need for natural hydrocolloids [12,13].

On the other hand, while the pulp has traditionally held lower commercial value compared to the seeds, and it is still more commonly used as a supplement in animal feed, its use in the food industry has been gaining attention [2,14].

Due to its high content of dietary fibre and antioxidant compounds, carob pulp has the potential to be used as a functional ingredient in food production [1]. Carob pulp stands out for being rich in sugars (>50%, mainly sucrose) and crude fibre (approximately 40%) and poor in protein (2–7%) [15,16], since most of it is found in the seed germ (55–65%) [13]. Due to its high sugar content, it can be used as a natural sweetener [15,16]. In addition, it is also used as a substitute for chocolate due to its similar colour and appearance [2]. Its affordability and absence of caffeine and theobromine, along with its high fibre and bioactive compound content, further bolsters its popularity for this purpose [2,17].

The aqueous extracts of carob pulp flour contain mainly dietary fibre, as well as a phenolic fraction consisting of water-soluble and insoluble tannins, flavonol glycosides and a high content of different forms of gallic acid [18]. These extracts have strong antioxidant activity, which makes them an interesting product for use in the development of functional foods [19]. However, the high tannin content is the cause of the bitter taste and makes its use for human consumption limited, as it causes excessive astringency [20].

The chemical composition of carob pulp flour varies depending on the cultivar, origin, environmental and climatic factors, and the harvest season [2,13,21,22]. The total harvested area of carob in the world during the last decade (2012–2021) is estimated at 74,229 ha, of which 66,944 ha (90%) are distributed among Spain, Portugal, Morocco, and Italy [23]. Spain is the leading producer country with 40,198 ha, which represents almost a quarter of the world’s production. Within Spain, the Balearic Islands represent 32% of total Spain production with 15,240 tn [24]. Several authors have evaluated the effect of cultivar in regions such as Portugal [25] and Egypt [22]. However, it is particularly intriguing to conduct such evaluations for the cultivars native to the Balearic Islands due to the economic significance of the crop in the region and the possibility of unique characteristics attributed to island cultivation. The insular environment may impart distinct qualities to the carob cultivars, making them potentially valuable resources with specific attributes not found elsewhere.

This study aimed to characterise the pulp of various carob cultivars from the Balearic Islands cultivated under controlled conditions in order to evaluate the impact of cultivar variation on its composition and assess its potential as a source of antioxidant compounds and functional properties, with the goal of incorporating these traits into the selection criteria for plant material in breeding programmes. The innovation of this research lies in the comprehensive assessment of the nutritional composition, functional attributes, and antioxidant activity of pulps from different carob cultivars.

## 2. Results and Discussion

### 2.1. Nutritional Analysis

A comprehensive nutritional analysis of the carob pulp powder (CPP) was performed. Since no significant differences (*p* > 0.05) were observed among cultivars for most parameters, Table 1 presents the mean, standard deviation, minimum and maximum values, along with the 95% confidence interval for these variables (moisture, energy value, carbohydrates, fibre, sugars, sucrose, glucose, ash, fats, butyric acid, and linoleic acid).

**Figure 1:**
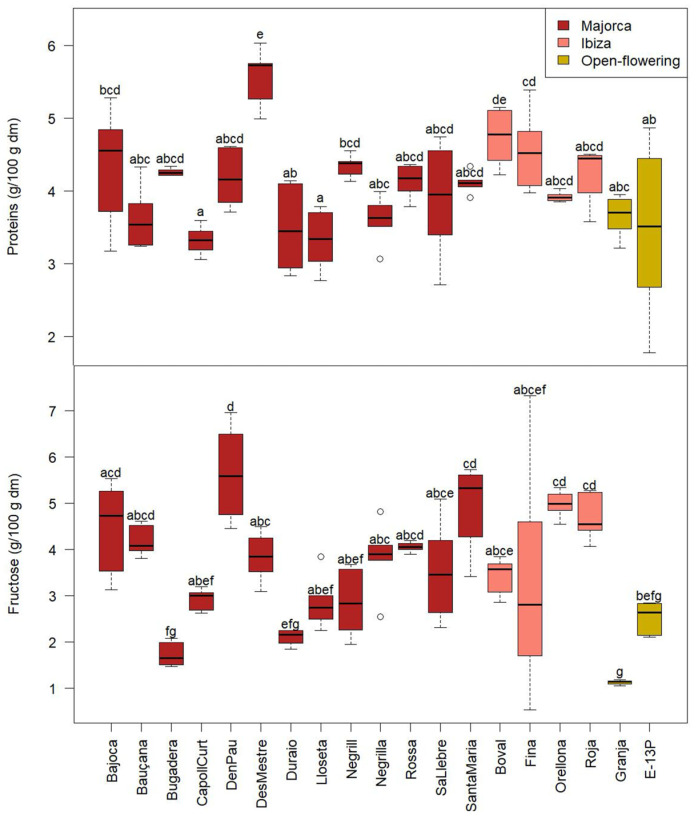
Protein and fructose content of the CPP from different cultivars from Majorca, Ibiza, and open-flowering selections. Different letters indicate significant differences (*p* < 0.05) among cultivars.

**Figure 2:**
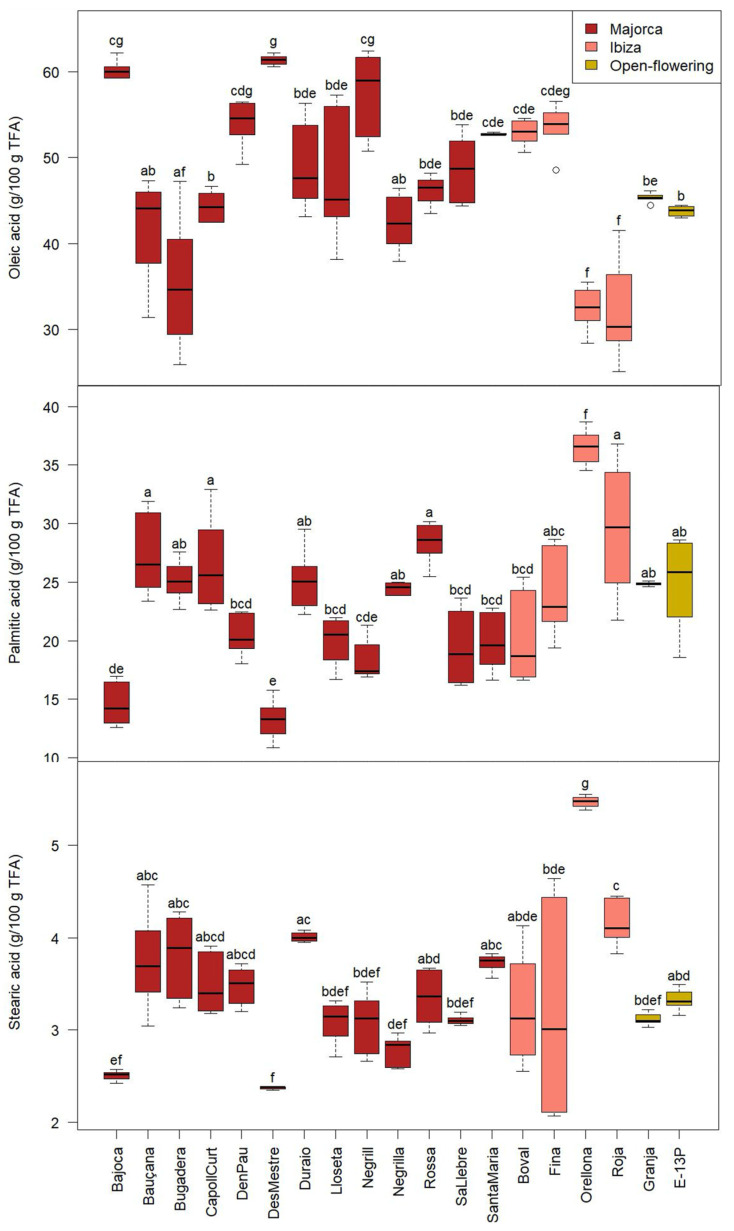
Oleic, palmitic, and stearic acids of the CPP from the different cultivars from Majorca, Ibiza, and open-flowering selections. TFA: total fatty acids. Different letters indicate significant differences (*p* < 0.05) among cultivars.

The parameters that showed significant differences (*p* < 0.05) among cultivars included protein and fructose content (Figure 1), as well as the fatty acid profile, specifically oleic, palmitic, and stearic acids (Figure 2).

To facilitate the comparison with other studies on CPP characterisation, Table 2 provides a summary of previously reported results in the literature.

Among the parameters that showed significant differences, protein content stood out (Figure 1). In particular, the ‘Lloseta’ and ‘Capoll Curt’ cultivars had significantly lower protein contents, at 3.3 ± 0.4 g/100 g dry matter (dm), on average, compared to the ‘Des Mestre’ cultivar, which reached 5.5 ± 0.4 g/100 g dm. It is worth noting that all carob pods were grown under identical climatic and geographic conditions. Therefore, the observed differences in protein content among cultivars are most likely due to genetic variation rather than environmental factors.

These values are consistent with those reported by Papaefstathiou et al. [10], who analysed carob pulp from Cyprus (5.0–5.4 g/100 g dm), and by Le Chami et al. [26], who characterised carob pulp powder (CPP) from Morocco, Spain, and Lebanon (4.7–5.8 g/100 g dm). However, they are slightly lower than the values reported by Yousif et al. [15] for carobs from Jordan (6.22 ± 0.38 g/100 g dm), Petkova et al. [27], who studied commercial CPP from Plovdiv, Bulgaria (5.9 ± 0.1 g/100 g dm), Simsek et al. [28], who evaluated wild carob pulp harvested in Turkey (6.1–9.1 g/100 g dm), and Kamal et al. [29], who analysed pulp from carobs cultivated in Egypt (6.74 g/100 g dm) (Table 2).

Interestingly, fructose content was also among the parameters that distinguished the cultivars. Specifically, the cultivars ’Granja’ and ’Bugadera’ exhibited significantly lower fructose levels (1.4 ± 0.4 g/100 g dm, on average) compared to ‘D’en Pau’, ’Santa Maria’ and ‘Orellona’ (5.2 ± 0.8 g/100 g dm, on average). Since all samples were grown under the same environmental conditions, this difference is most likely attributable to genotypic variation inherent to those cultivars, possibly reflecting metabolic traits selected in their region of origin (Ibiza for ‘Santa Maria’ and ‘Orellona’ and Majorca for ‘D’en Pau’).

Le Chami et al. [26] obtained values that varied from 7.8 to 11.1 g/100 g dm, observing lower values for CPP from Spain (7.8 g/100 g dm) than those from Lebanon (11.1 g/100 g dm). The values observed in this study were smaller than those reported by Biner et al. [30] (11.5 ± 2.7 g/100 g dm), who evaluated carob pulp from Turkey (4.2–19.0 g/100 g dm) and by Benković et al. [31] (17–19 g/100 g dm), who analysed Croatian carob pulp and similar to those observed by Petkova et al. [27], (4.2 ± 0.2 g/100 g dm).

On the other hand, the fatty acid profile also varied significantly (*p* < 0.05) among the cultivars (Figure 2). The oleic acid constituted the majority and exhibited significant differences among the cultivars, with ‘Orellona’ and ‘Roja’ displaying the lowest values (32.3 ± 4.4 g/100 g total fatty acids (TFA) on average), both originating from Ibiza, while ‘Des Mestre’ and ‘Bajoca’ cultivars from Majorca showcased the highest values (60.8 ± 1.0 g/100 g TFA, on average). Palmitic acid emerged as the second most abundant fatty acid, with ‘Orellona’ demonstrating a significantly (*p* < 0.05) higher content (36.6 ± 1.6 g/100 g TFA), while ‘Des Mestre’ exhibited the lowest values (13.3 ± 1.7 g/100 g TFA). Finally, stearic acid showed the same trend as palmitic acid, being significantly lower (*p* < 0.05) in ‘Des Mestre’ cultivar (2.4 ± 0.1 g/100 g TFA), and ‘Orellona’ showed the highest values (5.5 ± 0.1 g/100 g TFA). It is noteworthy that the cultivars ‘Bajoca’, ‘D’en Pau’, ‘Des Mestre’ and ‘Negrill’ exhibited notably higher levels of monounsaturated fatty acids (oleic acid), compared to the cultivars from Ibiza (‘Orellona’ and ‘Roja’), which were rich in stearic acid. Generally, these data coincide with those obtained by Kamal et al. [29], who reported 40.4 g/100 g TFA for oleic acid, 23.2 g/100 g TFA for linoleic acid, 11.1 g/100 g TFA for palmitic acid and 3.1 g/100 g TFA for stearic acid.

The moisture content of the CPP from the cultivars showed values with an average of 14.8 ± 1.7 g/100 g dm, similar to the 12.5 g/100 g dm reported by Yousif et al. [15] but higher than the values reported by several authors (4.6–9.3 g/100 g dm) [10,26,27,29]. It is important to highlight that, while previous studies presented results of toasted carob pulp flour, the present study produced fresh carob powder. This disparity may account for the elevated levels of humidity observed in our research findings. Therefore, all values compared with the literature are expressed on a dry matter (dm).

The energy value of the CPP from the different cultivars showed an average of 284.3 ± 9.5 kcal/100 g dm. Those values were lower than those previously reported (327–401 kcal/100 g dm) [10,27,29]. Therefore, generally, the cultivars analysed in this study presented a lower calorie content.

Carbohydrates were among the main components of CPP, with an average content of 60.7 ± 5.5 g/100 g dry matter. This observation is consistent with previous studies, which report carbohydrate contents ranging from 52 to 93 g/100 g dm [10,26,27,29].

On the other hand, the fibre content observed in this study showed an average of 31.7 ± 5.1 g/100 g dm, confirming that this flour is a good source of dietary fibre. These values exceed the 6 g/100 g threshold established for ‘high fibre’ nutritional claims, according to Regulation (EC) No. 1924/2006. The results are comparable to those reported by Papaefstathiou et al. [10], who found values between 30.3 and 37.3 g/100 g dm, and those reported by Le Chami et al. [26], who observed values of 37.3–38.4 g/100 g dm, but are significantly higher than the values reported by Yousif et al. [15] (12.4 g/100 g dm) and Kamal et al. [29] (7.70 g/100 g dm).

The total sugar content was 45.9 ± 8.1 g/100 g dm on average, the main one being sucrose, with an average value of 37.6 ± 9.0 g/100 g dm, followed by glucose with 4.7 ± 1.6 g/100 g dm. No significant differences (*p* > 0.05) were observed among cultivars for either glucose, sucrose, or total sugar content. The total sugar content is similar but lower than that reported by Biner et al. [30] (53.1 ± 9.3 g/100 g dm). The sucrose content was also similar to that reported in the literature (34–38 g/100 g dm) [27,30].

The ash content averaged 3.1 ± 0.3 g/100 g dm, representing a minor component of the CPP. These values are consistent with those reported by other authors, which range from 2.4 to 3.5 g/100 g dm [10,27,32].

The fat content was low with no significant (*p* > 0.05) differences among the cultivars (average of 0.7 ± 0.2 g/100 g dm). No significant differences were observed for linoleic acid, averaging 13.8 ± 2.4 g/100 g TFA. The butyric acid content obtained for carob cultivars (5.8 ± 3.3 g/100 g TFA), which is associated with the characteristic scent of carob, was higher than the figure reported by Ayaz et al. [33] of 1.3 g/100 g TFA.

When compared to other commonly used vegetable flours, CPP stands out for its significantly higher dietary fibre content, exceeding that of oat flour (2.3–8.5 g/100 g) [34] and chickpea flour (12.2 g/100 g) [35]. In terms of sugar content, CPP is comparable to dried dates, which contain between 44.4 and 79.8 g/100 g of total sugars [36], highlighting its potential use as a natural sweetener.

**Table 2:** Nutritional reported values in the literature for carob pulp powder (CPP) from different regions of the world.

Source	Origin	Moisture (g/100 g dm)	Energy (kcal/100 g dm)	Carbohydrates (g/100 g dm)	Protein (g/100 g dm)	Dietary Fibre (g/100 g dm)	Total Sugars (g/100 g dm)	Sucrose (g/100 g dm)	Fructose (g/100 g dm)	Glucose (g/100 g dm)	Ash (g/100 g dm)	TPC (mg GAE/g dm)	Antioxidant Activity (CUPRAC) (µmol TE/g dm)
Papaefstathiou et al. [10]	Cyprus	9.3	327–331	51.8–53.7	5.0–5.4	30.3–37.3	-	-	-	-	3.3–3.5	-	-
Kamal et al. [29]	Aswan, Egypt	5.5	366	75.92	6.74	7.70	-	-	-	-	3.4	-	-
Petkova et al. [27]	Plovdiv, Bulgaria	8.2 ± 0.02	401	92.5 ± 0.02	5.9 ± 0.1	-	-	34 ± 1	4.16 ± 0.21	4.25 ± 0.42	2.25 ± 0.02	8.10 ± 1.15	-
Yousif et al. [15]	Jordan	12.5	-	-	6.22 ± 0.38	12.4	-	-	-	-	3.1 ± 0.2	-	-
Simsek et al. [28]	Turkey	-	-	-	6.1–9.1	-	-	-	-	-	-	-	-
Biner et al. [30]	Turkey	-	-	-	-	-	53.1 ± 9.3	38.4 ± 7.6	11.5 ± 2.7	3.3 ± 1.6	-	-	-
Ayaz et al. [33]	Anatolia, Turkey	-	-	-	-	-	-	-	-	-	-	-	-
Benković et al. [31]	Croatia	-	-	-	-	-	-	35–38	17–19	18–22	-	15.58–17.96	34.78 ± 3.72
Fidan et al. [32]	Turkey	9.9	-	-	22.56	28.17	-	-	-	-	2.4–3.4	4.53 ± 0.08	-
Le Chami et al. [26]	Morocco	4.6 ± 0.8	-	90.5 ± 2.7	5.8 ± 1.8	37.3 ± 0.8	-	14.3 ± 6.7	8.1 ± 1.8	3.3 ± 1.8	3.4 ± 1.7	8.5	
	Spain	5.2 ± 1.3	-	92.3 ± 2.2	4.8 ± 1.8	37.9 ± 0.8	-	29.5 8.1	7.8 ± 1.6	3.0 ± 1.2	2.6 ± 0.7	12.7	
	Lebanon	6.8 ± 1.5	-	91.7 ± 2.7	4.7 ± 1.0	38.4 ± 1.0	-	27.4 10.3	11.1 ± 2.2	2.2 ± 1.6	3.2 ± 1.5	5.1	

### 2.2. pH and Acidity

**Figure 3:**
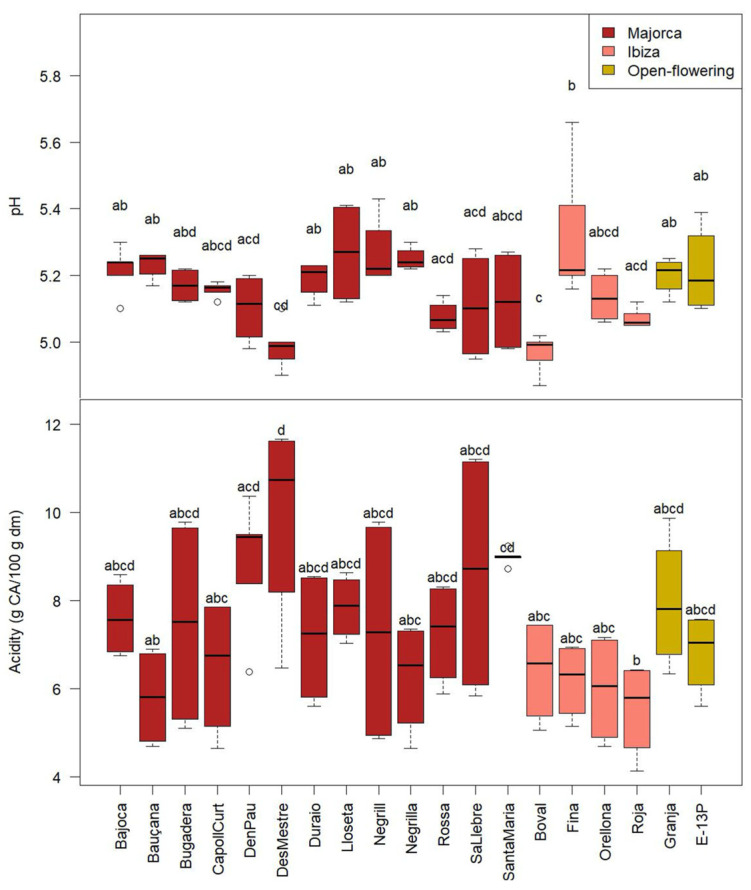
pH and acidity of the CPP from the different cultivars from Majorca, Ibiza, and open-flowering selections. CA: citric acid. Different letters indicate significant differences (*p* < 0.05) among cultivars.

Figure 3 shows the pH and acidity values of the carob pulp powder obtained from different carob cultivars. These parameters presented significant differences among cultivars (*p* < 0.05). Regarding the pH, ‘Fina’ was the cultivar with the highest average (5.3 ± 0.2), while the cultivars with the lowest pH were ‘Boval’ and ‘Des Mestre’ (average of 5.0 ± 0.1). Regarding the acidity, ‘Des Mestre’ was the cultivar with the highest value of this parameter (9.9 ± 0.1 g of citric acid (CA)/100 g dm). ‘Roja’ was the cultivar with the lowest acidity (5.5 g CA/100 g dm), followed by ‘Bauçana’ (5.8 ± 1.1 g CA/g dm). Differences in these parameters could indicate variations in flavour, as higher acidity is generally associated with a more sour taste, whereas lower acidity and higher pH typically result in a milder and sweeter flavour profile [37].

In this study, the pH of the cultivars varied in the range 5.0–5.3, lower than the value obtained in the study by Yousif et al. [15], in which an average of 6.0 was obtained for CPP obtained with different processing conditions of roasting. The values observed in this study are similar to those mentioned by Tetik et al. [3], who obtained a pH of 5.4 in wild carob and 5.3 in grafted carob from Turkey.

The relatively low pH observed in the CPP can be attributed to the high content of organic acids present in the material. Polat et al. [38] analysed carob pulp from three Turkish genotypes and reported the main organic acids as succinic, citric, and malic acid, followed by smaller amounts of oxalic, ascorbic, and fumaric acid. These organic acids contribute significantly to the acidity and thus the relatively low pH of CPP.

### 2.3. Soluble Solids Content (SSolids)

**Figure 4:**
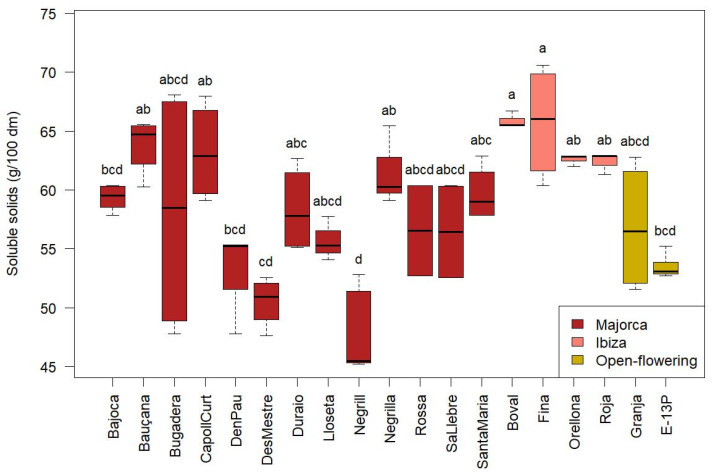
Soluble solids of the CPP from the different cultivars from Majorca, Ibiza, and open-flowering selections. Different letters indicate significant differences (*p* < 0.05) among cultivars.

SSolids content showed significant differences among cultivars (*p* < 0.05), ranging from 45.2 to 70.6 g/100 g dm. As illustrated in Figure 4, the ‘Fina’ cultivar exhibited the highest average concentration (65.7 ± 4.7 g/100 g dm), while ‘Negrill’ showed the lowest (47.6 ± 3.5 g/100 g dm). Overall, the Ibiza cultivars (highlighted in pink in Figure 4) generally displayed higher SSolids content.

Considering that CPP presented values of sugar content of 45.9 ± 8.1 g/100 g dm and of fibre 31.7 ± 5.1 g/100 g dm, the high SSolids values found—especially in cultivars like ‘Fina’—are likely driven largely by sugars, with soluble fibres contributing a smaller portion.

The values reported by Tetik et al. [3] ranged from 59.4 to 64.1 g/100 g, which are comparable to those found in the present study. However, their results were expressed on a wet basis, and no information regarding moisture content was provided, making direct comparison difficult.

### 2.4. Total Phenolic and Tannin Content and Antioxidant Activity

**Figure 5:**
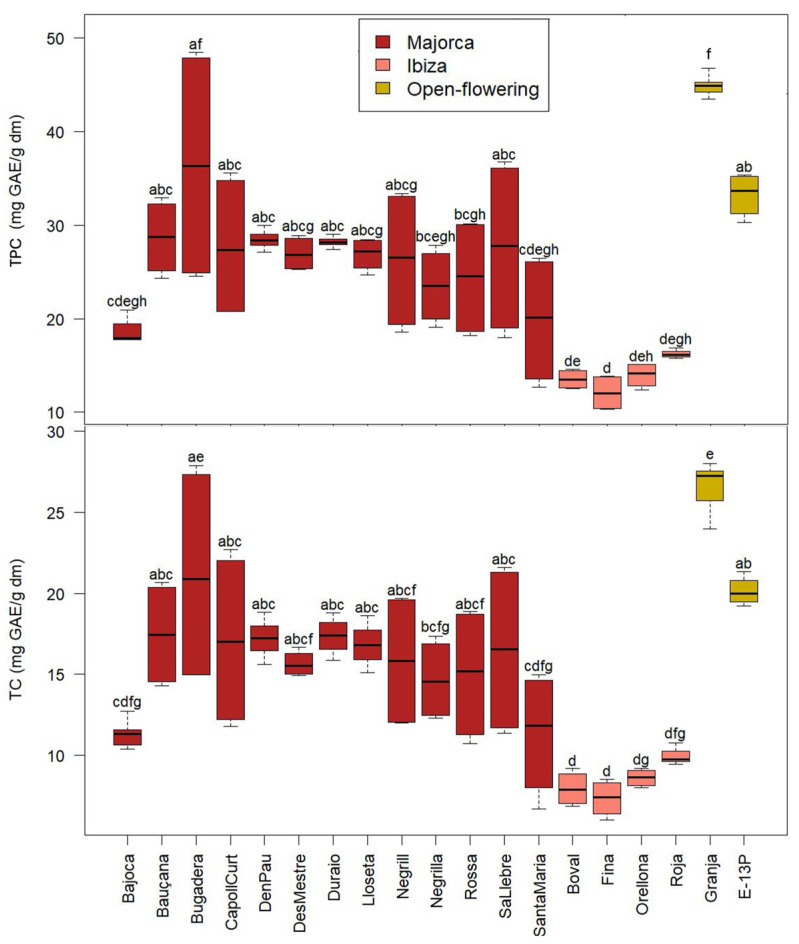
Total phenolic content (TPC) and tannin content (TC) of the CPP from the different cultivars from Majorca, Ibiza, and open-flowering selections. Different letters indicate significant differences (*p* < 0.05) among cultivars.

**Figure 6:**
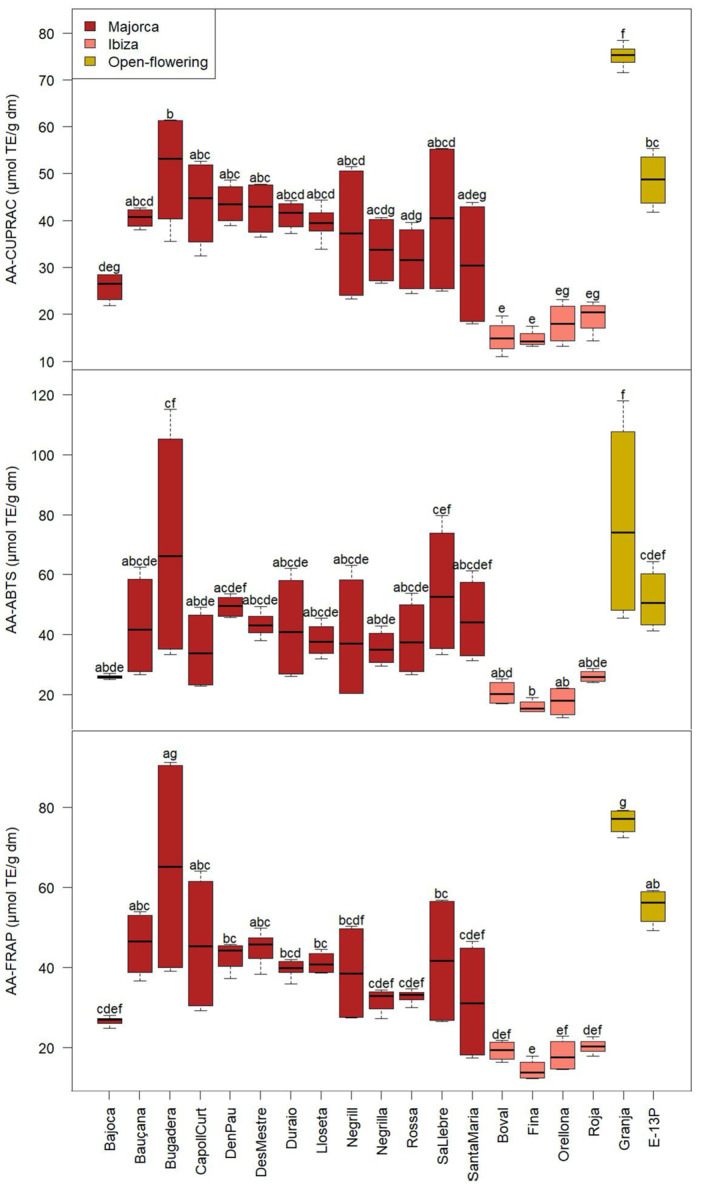
Antioxidant activity according to CUPRAC, FRAP, and ABTS assays of the CPP in the different cultivars from Majorca, Ibiza, and open-flowering selections. Different letters indicate significant differences (*p* < 0.05) among cultivars.

The results for total phenolic content (TPC) and total tannin content (TC) are presented in Figure 5, and those of antioxidant activity (AA) are presented in Figure 6. Taking into account that all carob pulp samples were collected at the same geographical location and vintages (same edafoclimatic characteristics and cultivation practices), differences detected in the polyphenol content may be mainly attributed to the intrinsic properties of each carob cultivar.

Regarding the TPC, values varied from 10.4 to 48.5 mg GAE/g dm, exhibiting significant differences among cultivars. The two open-flowering selections presented the highest concentration, ‘Granja’ being the one with the highest average of TPC (45.1 ± 1.1 mg GAE/g dm), followed by ‘E-13P’ (33.2 ± 2.3 mg GAE/g dm). Interestingly, the lower TPC was exhibited by the carob cultivars from Ibiza Island: ‘Fina’, ‘Boval’, ‘Orellona’, and ‘Roja’ (average of all Ibiza cultivars of 14.0 ± 2.0 mg GAE/g dm).

Due to the different carob sample treatments or extraction methodologies used to evaluate the TPC, data in the literature vary notably. The values of the present study were higher than those reported by Benković et al. [31] in Croatian carob pulp (17.4–19.6 mg GAE/g dm) and those obtained by Petkova et al. [26] in carob flour from the Bulgarian market (8.1 mg GAE/g dm) (Table 2). Le Chami et al. [26] reported values for CPP from Spain of 12.7 mg GAE/g dm, higher than those of Morocco and Lebanon (8.5 and 5.1 mg GAE/g dm, respectively), highlighting the influence of the geographical origin of the samples.

Regarding the TC, the results were in concordance with the TPC. Results of the TC for CPP ranged from 6.0 to 28.0 mg GAE/g dm. ‘Granja’ (26.6 ± 1.5 mg GAE/g dm), followed by ‘Bugadera’ (21.2 ± 6.7 mg GAE/g dm) and ‘E-13P (H2-12)’ (21.1 ± 0.8 mg GAE/g dm), were those with the highest tannin content. On the other hand, in agreement with the TPC, the cultivars from Ibiza Island showed the lowest tannin content (average of all cultivars from Ibiza 8.5 ± 1.3 mg GAE/g dm).

Cultivars with higher tannin content represent a valuable source of antioxidant activity; however, their high levels may also contribute to an increased astringency, potentially affecting sensory acceptability [38].

The CUPRAC and FRAP assays were used to measure the capability to capture copper and iron, respectively, while the ABTS assay was conducted to assess the ability to neutralise free radicals. Results of this analysis are depicted in Figure 5.

The CUPRAC assay yielded values varying from 11.0 to 78.4 µmol TE/g dm. The value reported by Fidan et al. [32] was within the range observed in this study (34.8 µmol TE/g dm). The cultivar with the highest antioxidant activity according to the CUPRAC assay was ‘Granja’ (75.1 ± 2.4 µmol TE/g dm), and those cultivars with the lowest antioxidant activity according to CUPRAC were those from Ibiza (average of 16.9 ± 3.9 µmol TE/g dm). As can be seen in Figure 5, both FRAP and ABTS followed the same trend as CUPRAC.

Regarding the antioxidant activity according to the FRAP assay, the values varied from 12.35 to 91.26 µmol TE/g dm. These values were lower than those reported by Fidan et al. [32] for carob pulp flour from Turkey (109.1 µmol TE/g dm).

According to the ABTS assay, the antioxidant activity varied from 12.5 to 118.0 µmol TE/g dm; these values are lower than those reported by Benković et al. [1] (168.0–171.5 µmol TE/g dm) and similar to the value reported by Fidan et al. [32] (64.7 µmol TE/g dm).

A greater variability was observed among the Majorcan cultivars in terms of TPC, TC and antioxidant activity, as evidenced by their noticeably higher standard deviations compared to the more uniform values observed in the Ibizan and open-flowering cultivars. This pattern suggests that the Majorcan group may possess broader genetic diversity.

The Pearson coefficients were determined to evaluate the degree of association between the TPC, the TC and the antioxidant activity determined by the ABTS, CUPRAC and FRAP assays. These coefficients were high and positive (0.80–0.97) in all cases. The highest correlation coefficients were observed between the TPC and the TC, with the antioxidant activity measured by the FRAP assay (0.97 and 0.96, respectively). Several authors have reported good correlations between the antioxidant activity and the polyphenol content [39,40].

### 2.5. Colour

**Figure 7:**
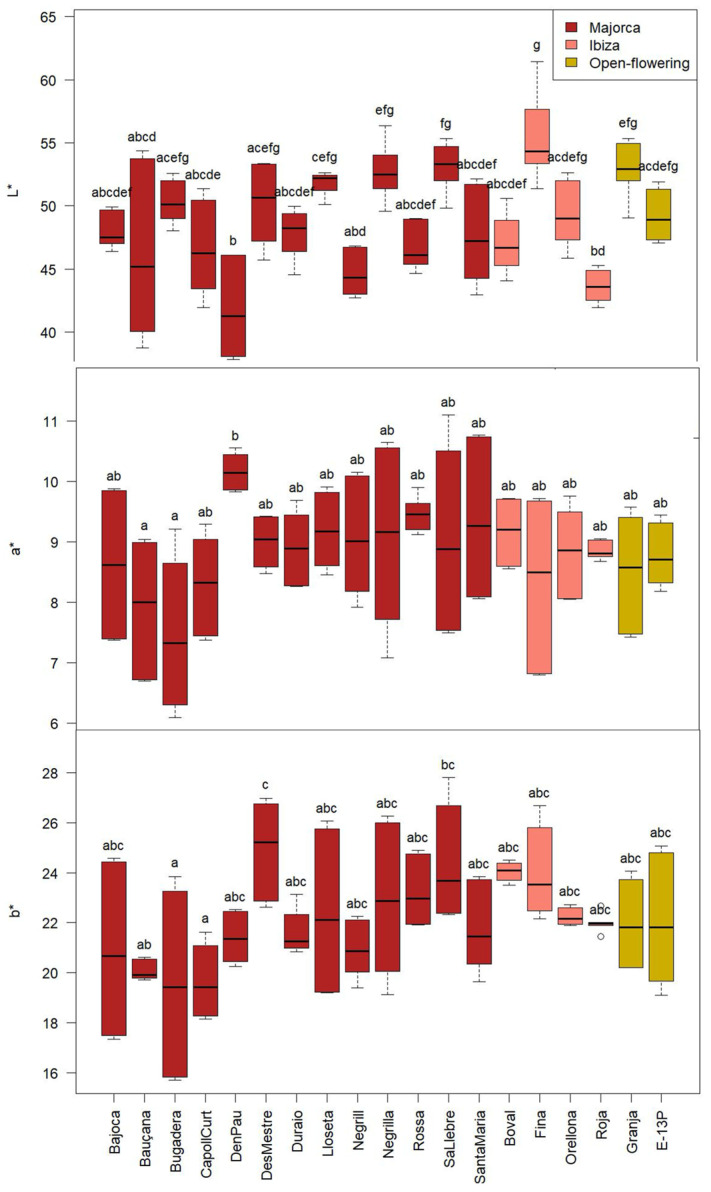
CIELab parameters of the CPP from the different cultivars from Majorca, Ibiza, and open-flowering selections. Different letters indicate significant differences (*p* < 0.05) among cultivars.

Colour is a key quality attribute in vegetable-based food products, strongly influencing consumer preference and perception of freshness or ripeness. Statistically significant differences (*p* < 0.05) were observed among cultivars for all CIELab colour parameters, as shown in Figure 7. The lightness (L*) values ranged from 38 to 61, with ‘D’en Pau’ exhibiting the lowest average (41 ± 4) and ‘Fina’ the highest (55 ± 4). The a* and b* parameters, which represent the red-green and yellow-blue colour axes, respectively, ranged from 6 to 11 and 16 to 28. ‘D’en Pau’ had the highest a* (10 ± 2), and ‘Des Mestre’ showed the highest b* (25 ± 2).

**Figure 8:**
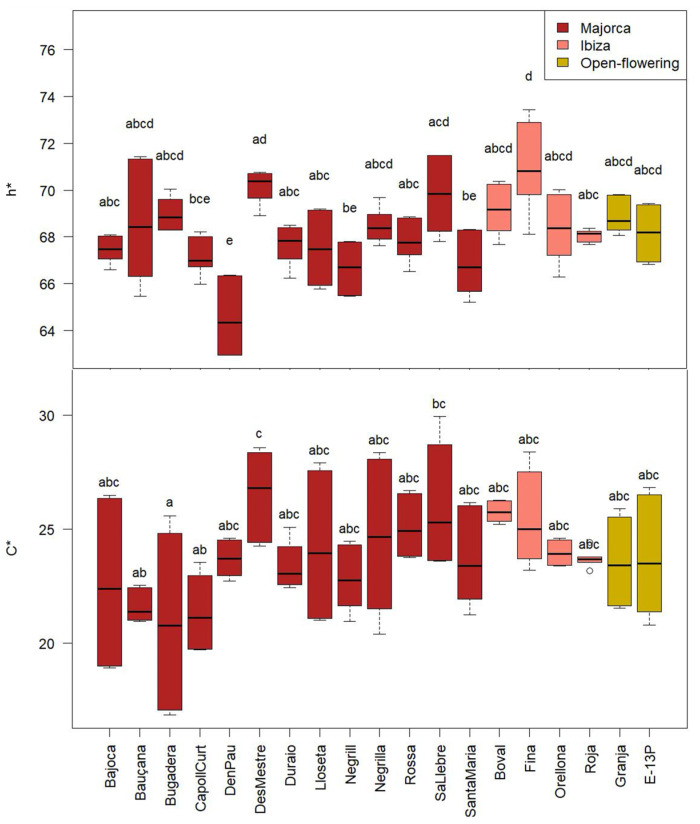
Croma (C*) and hue (h*) parameters of the CPP from the different cultivars from Majorca, Ibiza, and open-flowering selections. Different letters indicate significant differences (*p* < 0.05) among cultivars.

Chroma (C*), which reflects colour intensity or saturation, varied between 17 and 30, with ‘Bugadera’ showing the lowest value (21 ± 4). Hue angle (h*), which indicates the type of colour perceived (e.g., red, yellow), ranged from 63° to 73°. This range falls within the yellowish-orange region of the spectrum, but the low lightness and chroma of the samples result in an overall brown appearance. ‘Fina’ displayed the highest h* angle (71 ± 2°), suggesting a shift towards more yellow tones. These differences in colour attribute may reflect underlying differences in pigment composition among cultivars, with potential implications for both consumer appeal and nutritional value (Figure 8).

These variations among the cultivars were, as anticipated, given the distinct polyphenol profiles influenced by the presence of flavonoids, contributing to the yellowish colour, and the proportion of proanthocyanidins, which give rise to the brown colour [41].

There is a lack of studies quantifying the colour parameters of CPP; however, Eldeeb et al. [42] reported comparable, though slightly higher, values for L* (69). The a* (9) and b* (22) values were within the range observed in the present study.

It could be interesting to compare values with cocoa pulp colour, as carob pulp is often used as a substitute for cocoa. Thus, carob pulp exhibited a reddish-brown colour in all cultivars, similar to that of cocoa and may be a desirable attribute for use as a dye in other pastry products.

In comparison with cacao pulp colour, Reges et al. [43] reported L* values varying between 60 and 75, similar to the carob pulp values obtained in the presented study. The h* is situated between 69 and 81°, closer to the h* values of carob pulp reported in our study.

### 2.6. Functional Properties

**Figure 9:**
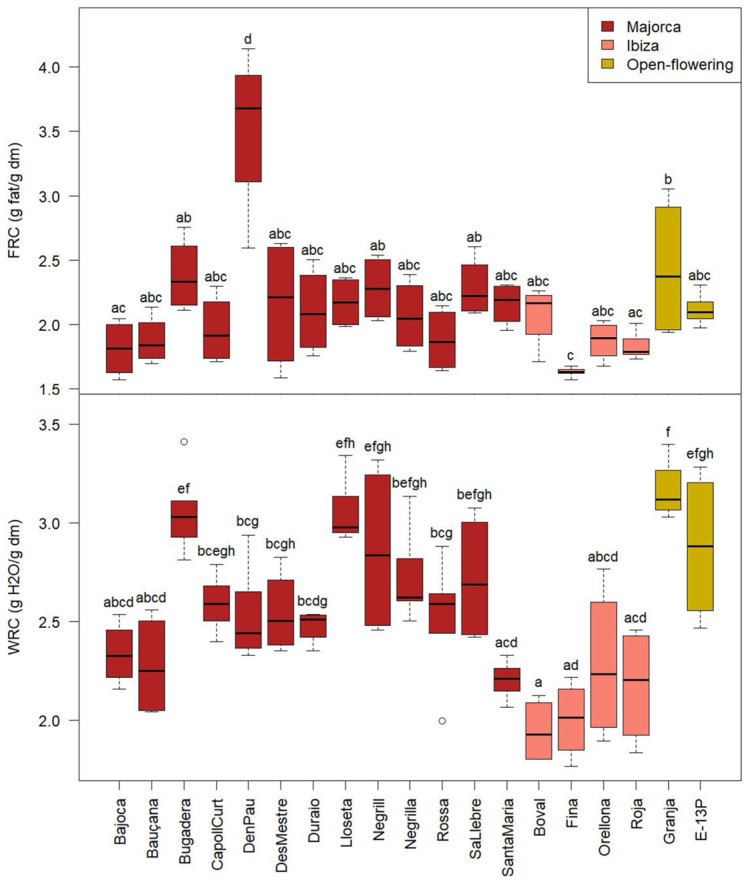
Fat adsorption capacity (FRC) and water retention capacity (WRC) of the CPP from the different cultivars from Majorca, Ibiza, and open-flowering selections. Different letters indicate significant differences (*p* < 0.05) among cultivars.

The functional properties of potential food ingredients, such as fat retention capacity (FRC) and water retention capacity (WRC), are closely linked to the physicochemical characteristics of cell wall polysaccharides. WRC, primarily associated with insoluble dietary fibre, plays a crucial role in preventing and managing various intestinal disorders by increasing faecal bulk and reducing gastrointestinal transit time. From a food technological perspective, dietary fibre with a high FRC enables the stabilisation of fat in emulsion-based products. Conversely, ingredients with high WRC can serve as a functional ingredient to prevent syneresis and alter the viscosity and texture of certain formulated foods [43]. The values obtained for WRC and FRC of CPP from different cultivars are shown in Figure 9.

FRC results ranged from 1.6 to 4.1 g fat/g dm. The CPP exhibited significant differences in FRC values among cultivars (*p* < 0.05). ‘D’en Pau’ (3.5 ± 0.6 g fat/g dm) was the cultivar that retained more fat on its surface. This suggests that ‘D’en Pau’ may be particularly suitable for food applications where fat binding is desirable. This includes products such as meat analogues, bakery items, or emulsified systems, where improved fat retention can enhance texture, flavour stability, and reduce fat loss during processing or cooking.

WRC values ranged from 1.8 to 3.4 g H_2_O/g dm, exhibiting significant differences among cultivars. ‘Granja’ was the cultivar with the highest WRC (3.2 ± 0.1 g H_2_O/g dm). On the other hand, ‘Boval’, and ‘Fina’ (2.0 ± 0.2 g H_2_O/g on average) were the cultivars with the lowest capacity to retain water. In general, the cultivars studied have a greater capacity to retain water than to adsorb fats. A high WRC in an ingredient can help prevent liquid loss during processing or in the final product (syneresis) [44].

The Balearic cultivars showed higher water retention capacity and lower fat adsorption capacity than those studied by Petkova et al. [27]. These authors reported higher values for fat adsorption than for water retention, which is opposite to that observed in the present study. However, it should be noted that in that case, the samples were toasted carob flours, which could have changed their properties. The WRC exhibited by the cultivars was comparable to that of other flours, such as pea flour (1.2–2.8 g H_2_O/g), which is widely used in the food industry for its functional properties [44]. It is still similar but lower than the values observed for oat flour (2.8–3.4 g/g) [45].

### 2.7. Principal Component Analyses

Principal Component Analysis (PCA), performed to reduce the dimensionality of the data while preserving as much variability as possible, was carried out considering only those parameters that showed statistically significant differences (*p* < 0.05) among cultivars. Additionally, some variables with very high inter-correlation were excluded to avoid redundancy; specifically, only CUPRAC, with the highest significance, was included among the antioxidant-related parameters (TPC, TC, and AA measured by using the ABTS, CUPRAC, and FRAP assays). In the case of colour parameters, L* and b* were included in the PCA because of their higher significance. Similar to the fatty acids, only oleic and stearic acids were considered. Thus, 12 variables were included for the PCA (protein, fructose, oleic acid and stearic acid contents, pH, acidity, soluble solids content, AA—according to the CUPRAC assay—L*, b*, WRC, and FRC).

**Figure 10:**
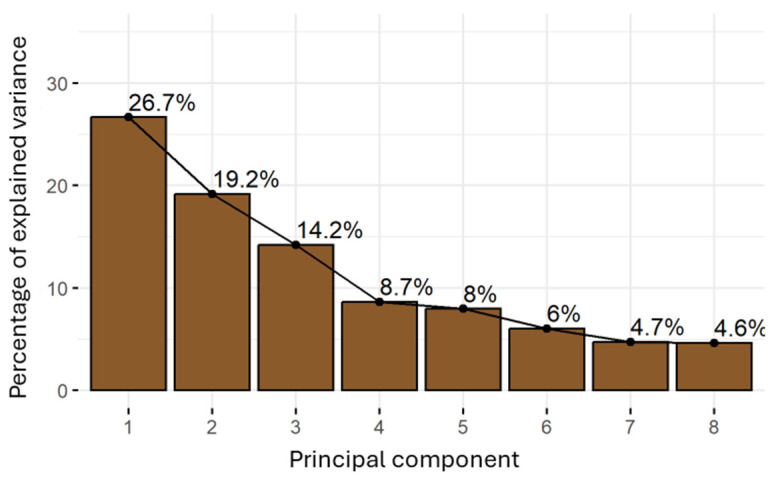
Percentage of the explained variance of each principal component of the PCA for the CPP from the different cultivars from Majorca, Ibiza, and open-flowering selections.

**Figure 11:**
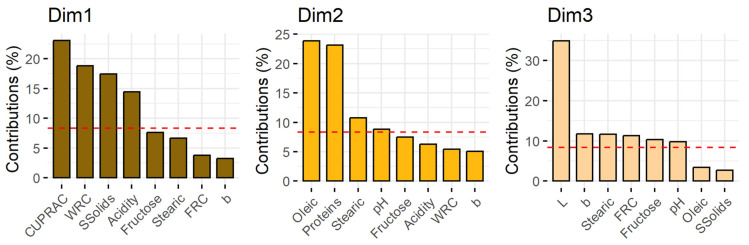
Contribution of the variables to the first three principal components of the PCA (Dim1, Dim2, and Dim3) for the CPP from the different cultivars from Majorca, Ibiza, and open-flowering selections. The red dashed line indicates the expected average contribution.

Figure 10 shows the explained variance and the cumulative percentage of explained variance for the first eight principal components. It can be observed that the first three principal components explained 60.1% of the variance. Figure 11 shows the contribution of each variable to the first three principal components (Dim1, Dim2, and Dim3). The red dashed line on the figure indicates the expected average contribution. For a given component, a variable with a contribution above this threshold can be considered important in contributing to that component. The first principal component (Dim1) was mainly influenced by the AA (CUPRAC), WRC, SSolids, and acidity, reflecting differences in antioxidant and functional properties. Dim2 is primarily driven by oleic acid, protein, and stearic acid, highlighting compositional variations in fats and proteins. Dim3 is mostly affected by lightness (L*), followed by b*, indicating that this component is largely associated with the colour.

**Figure 12:**
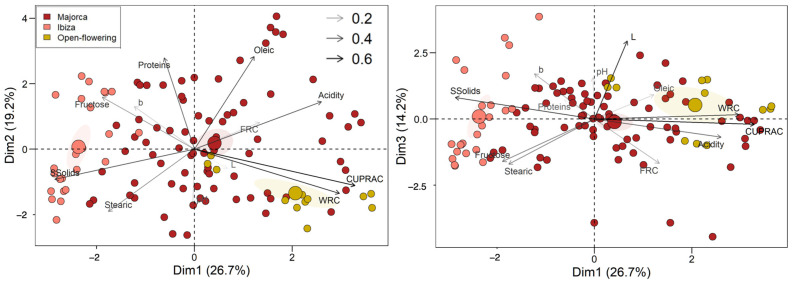
Representation of the variables and samples in the Dim2 vs. Dim1 (**left**) and Dim3 vs. Dim1 (**right**) coordinates of the CPP from the different cultivars from Majorca, Ibiza, and open-flowering selections.

In Figure 12, the variables and samples are represented in the Dim1-Dim2 (left) and Dim1-Dim3 (right) coordinate systems. The colour of the vectors reflects their importance in the model, with darker colours indicating the strongest contributors. This plot illustrates the contribution and direction of each parameter to the components: Dim1 is mainly associated positively with antioxidant activity and WCR and negatively with SSolids; Dim2 is influenced positively mainly by oleic acid and proteins and negatively related to pH and stearic acid. Finally, Dim3 is mostly associated positively with L* and negatively with FRC. As can be seen in the Dim1-Dim2 coordinate system, Ibiza cultivars (‘Boval’, ‘Orellona’, ‘Rossa’, ‘Roja’, and ‘Fina’) cluster in the left quadrant (mainly in the lower one), associated with higher levels of SSolids, fructose and stearic acid and lower levels of antioxidant activity, WRC and acidity. In contrast, open flower selection cultivars (‘Granja’ and ‘E-13P’) are located mainly in the right quadrant, strongly associated with high antioxidant activity and WRC and low SSolids and fructose. This suggests that open flowering cultivars may allocate more metabolic resources towards the synthesis of antioxidant compounds rather than sugar accumulation, reflecting differences in their genetic background and selection history. Majorca cultivars are more widely distributed, reflecting a broader range of physicochemical characteristics. In the Dim1-Dim3 plot, an even greater separation of the Ibizan cultivars is observed. This indicates that not only the variables involved in Dim2 differentiate this set of samples, but also the brightness (L*) of the CPP. These results suggest that the genetic background of the cultivars plays a significant role in determining their physicochemical profiles, as all samples were grown and harvested under identical environmental and agronomic conditions.

## 3. Materials and Methods

The carob samples were obtained from a germplasm bank of the Institute for Agri-food and Fishing Research and Training of the Balearic Islands located in 39.737767, 3.177965-39°44′16.0″ N 3°10′40.7″ E (Majorca, Balearic Islands). All carob cultivars were planted in 2014, shared the same yard location, cultivation system, climate, soil type and harvesting during the experiment.

The carob beans were harvested by hand when they reached the RS5 ripening stage [46] during two different seasons (2021 and 2022). The meteorological conditions in the experimental yard showed temperature ranges of 16.70–40.42 °C in 2021 and 17.12–40.48 °C in 2022, with 3 and 5 days exceeding 38 °C in each respective year. The accumulated precipitation was 576.16 mm in 2021 and 622.16 mm in 2022.

**Figure 13:**
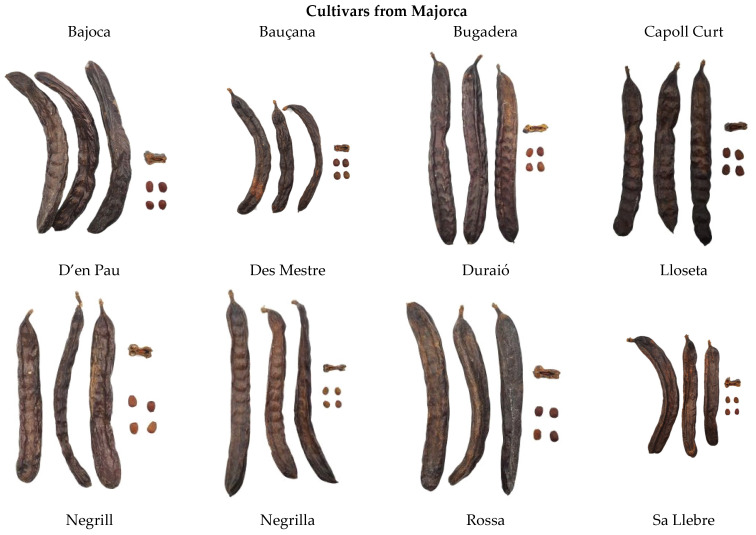

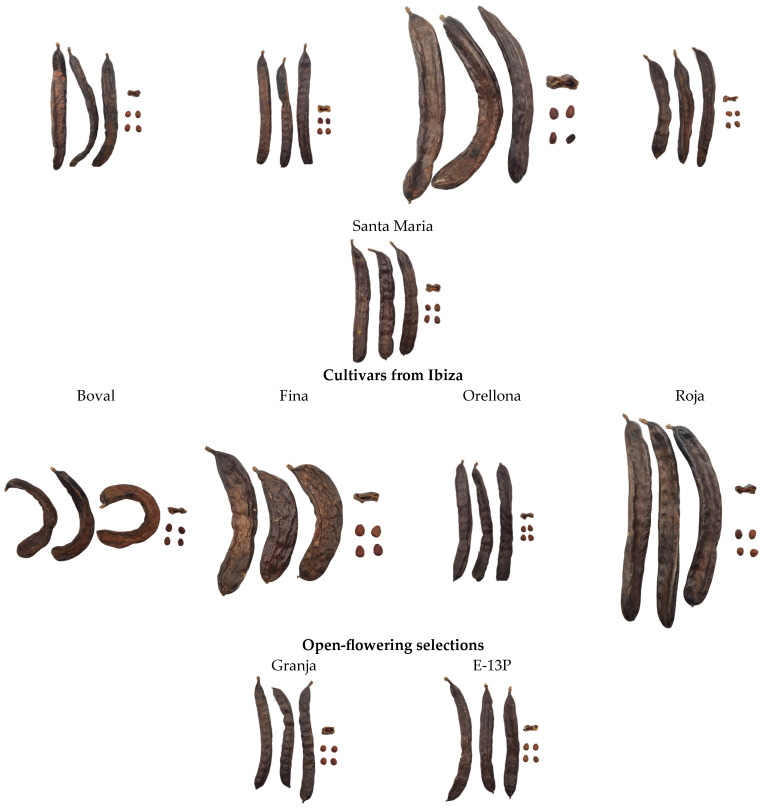
Photographs of the pods, seeds, and pulp from the 19 carob (*Ceratonia siliqua* L.) cultivars evaluated in this study.

For this study, 19 cultivated cultivars were used; there were 13 from Majorca Island (‘Bajoca’, ‘Bauçana’, ‘Bugadera’, ‘Capoll curt’, ‘D’en Pau’, ‘Des Mestre’, ‘Duraió’, ‘Lloseta’, ‘Negrill’, ‘Negrilla’, ‘Rossa’, ‘Sa Llebre’ and ‘Santa Maria’); 4 from Ibiza island (‘Boval’, ‘Fina’, ‘Orellona’, ‘Roja’) and 2 open-flowering selections (‘E-13P’ also known as ‘H2-12’) and ‘Granja’). In the last case, both cultivars were selected by agronomy parameters from the experimental plot of the Institute for Agri-food and Fishing Research and Training of the Balearic Islands, from a population of carob plants that were reproduced through natural, uncontrolled pollination, maintaining genetic diversity and adaptability. A photograph of each cultivar (pod, pulp and seeds) can be seen in Figure 13.

### 3.1. Preparation of Carob Pulp Powder

Twenty representative carob beans of each cultivar were selected, and the external dirt was cleaned. The carob beans were crushed, and the pulp was separated from the carob seed. The pulp was ground with a mill (IKA M20 Universal Mill, Königswinter, Germany) and sieved to obtain a homogeneous powder sample with a particle size lower than 450 µm. This powder will be called “carob pulp powder” (CPP). All analytical results are reported as the average of the two harvesting seasons (2021 and 2022). Samples were placed in hermetically sealed polyethylene bags and stored in a temperature-controlled chamber at 5 °C and protected from light.

### 3.2. Nutritional Analysis

The nutritional composition of the CPP was determined by AGROLAB IBÉRICA, located in Burgos, Spain, which is accredited under UNE-EN ISO/IEC 17025:2017 [47]. The analysis complies with Regulation (EC) No. 1169/2011. For this analysis, 100 g of ground sample was supplied per cultivar and year studied. Analyses were carried out at least in duplicate. Sugars were quantified by ion chromatography with a conductivity detector after aqueous extraction and filtration; total fat content was determined via Soxhlet ether extraction; fatty acid composition was analysed by gas chromatography; and protein content was determined using the Kjeldahl method (N × 6.25). The ashes and moisture content were determined gravimetrically according to the AOAC 923.03 and ISO 2483-1973 [48,49], respectively, and the fibre was determined according to the AOAC 985.29 [50]. Moisture content was used to calculate the composition in dry matter (dm).

### 3.3. Reagent

For the following analysis, the reagents used included sodium hydroxide (pellets) and methanol (analytical grade), both from Scharlau (Barcelona, Spain). The Folin–Ciocalteu reagent and gallic acid monohydrate were also obtained from Scharlau. Trolox (97% purity), from the Acros Organics brand (Geel, Belgium), was purchased in Mallorca, Spain. Neocuproine, 2,2′-azino-bis(3-ethylbenzothiazoline-6-sulphonic acid) (ABTS), and potassium bromide were purchased from Sigma-Aldrich (Madrid, Spain). Iron(III) chloride hexahydrate and potassium persulfate were acquired from Scharlau. Finally, 2,4,6-tris(2-pyridyl)-s-triazine (TPTZ), from the Fluka Analytical brand (Buchs, Switzerland), was also obtained in Mallorca, Spain.

### 3.4. pH and Acidity

The pH determination was performed from 5 g of sample added to 20 mL of distilled water. The pH was measured while stirring with a pH-meter (pH 25+, Crison, Barcelona, Spain) until reaching a stable pH value. To calculate acidity, 0.1 N NaOH was added until pH 8.1 [51]. The acidity results are expressed as g of citric acid (CA)/100 g dm of CPP. At least 4 values for each cultivar were obtained.

### 3.5. Soluble Solids Content

The soluble solids (SSolids) content was determined by measuring the °Brix of the samples. For this, 2.5 g of carob powder were weighed, and 25 mL of distilled water were added. It was left stirring at 40 °C for 15 min. Filtration was carried out under reduced pressure. The resulting liquid was allowed to cool to 20 °C and the °Brix was measured with a refractometer (ZUZI, Series 300, model 50301130, Valencia, Spain). At least 4 values for each cultivar were obtained.

### 3.6. Total Polyphenol Content, Tannic Content, and Antioxidants

#### 3.6.1. Extraction

For the analysis of the total polyphenol and tannin contents, and the antioxidant activity, an extraction was first carried out from the carob powder samples following the protocol described by Červenka et al. [52], with some modifications. For this, 1.5 g of sample was weighed, and 10 mL of MeOH/H_2_O (50:50) were added. This was vortexed for 3 min and allowed to shake for 1 h. Afterwards, the samples were centrifuged at 2500 rpm for 10 min and the liquid part was separated. To the remaining solid part, 10 mL of Acetone/H_2_O (70:30) were added and vortexed again for 3 min and shaken for 1 h. Then, the liquid separated above was added, and the whole was centrifuged together at 5000 rpm for 10 min. Finally, the sample was filtered through 0.45 µm PTFE filters and stored at −20 °C until analysis. At least 4 values for each cultivar were obtained.

#### 3.6.2. Total Phenolic Content (TPC)

The TPC of the different cultivars of carob powder was estimated by Folin–Ciocalteu method, as described by Eim et al. [53]. The polyphenol content was determined by mixing 10 µL of the sample extract, 5 µL of the Folin-Ciocalteu reagent and 95 µL of distilled water. This was allowed to stand for 5 min and 80 µL of 7.5% sodium carbonate was added. Absorbance was measured at 745 nm for 30 min every 5 min using a UV/Vis/NIR spectrophotometer (Thermo Scientific Multiskan Spectrum, Vantaa, Finland) and processing the data with SkanIt 2.4.2 Software. Polyphenol content was calculated from a calibration curve using gallic acid as a standard (0.025–0.3 mg/mL). Results were expressed as mg of gallic acid equivalent (GAE) per g of carob powder (mg GAE/g CPP dm), from two measurements.

#### 3.6.3. Tannin Content (TC)

To quantify the total TC, protein precipitation was performed with bovine serum albumin (BSA) as described by Ricco et al. [54]. To prepare this solution, 0.2 M pH 5.0 acetate buffer was prepared by diluting 1.64 g of CH_3_COONa in 100 mL of water. The pH was adjusted with acetic acid or sodium hydroxide. Then, 0.99 g of NaCl was diluted with 100 mL of acetate buffer. Finally, 100 mL of BSA was added to 100 mL of the prepared solution. In a tube, 1 mL of the sample extract and 1 mL of the BSA solution were added. It was left to rest for 15 min and centrifuged at 4200 rpm for 10 min. With the resulting supernatant, the polyphenol content was again determined following the Folin-Ciocalteu protocol. To obtain the results of total tannins, the initial polyphenol content was subtracted from the polyphenol content after precipitation with BSA, which allowed knowing the phenolic content that behaves as tannins.

#### 3.6.4. Antioxidant Activity

Antioxidant compounds can act through different mechanisms, and there is no single analytical assay that can evaluate the antioxidant activity of the sample. Therefore, different antioxidant activity analyses have to be performed to obtain more precise data on the antioxidant properties of the sample [55]. In this study, the ABTS, CUPRAC and FRAP assays were performed.

All spectrophotometric determinations were performed with a UV/Vis/NIR spectrophotometer (Thermo Scientific Multiskan Spectrum, Vantaa, Finland) using microplaques and processing the data with SkanIt 2.4.2 Software. All data, reported as trolox equivalents (TE), were calculated from a calibration curve ranging from 0.05 to 0.35 mg trolox/mL. Results were expressed as µmol TE/g dm.

The ABTS assay was conducted by discolouration of the radical cation 2,2′-azino-bis-(3-ethylbenzothiazoline-6-sulfonic acid) (ABTS•+), based on a slightly modified version of the experimental procedure described by González-Centeno et al. [39]. The ABTS reagent was prepared by first mixing equal volumes (1:1) of two solutions: one containing 7 mM ABTS radical cation and the other containing 2.45 mM potassium persulfate. Then, 8 mL of this mixture was diluted with a solution of ethanol and water (25:75 ratio) to a final volume of 100 mL. First, 190 µL of this reagent was introduced and incubated for 10 min at 25 °C and the absorbance was measured. After the incubation time, the sample was added, and absorbance readings were taken at 734 nm every 5 min for 30 min, values of 30 min were used since at this point the reaction was completed.

For the CUPRAC assay, the cupric reducing antioxidant activity was determined according to the method of González-Centeno et al. [40]. CUPRAC reagent was prepared from a combination of 10 mM Cu(II) aqueous solution, 7.5 mM neocuproine solution and 1 M ammonium acetate buffer (1:1:1 *v*/*v*). First, 190 µL of the reagent was added and it was left to incubate for 10 min at 25 °C and the absorbance was measured. Then, 10 µL of the sample was added and absorbances were measured at 450 nm every 5 min for 30 min, values of 30 min were used since at this point the reaction was completed.

For the FRAP assay, the ferric reducing antioxidant power method is based on the absorbance increase at 593 nm due to the formation of 2,4,6-Tri-(2-pyridyl)-s-triazine complexes with iron (II) in the presence of a reductive agent [56]. The protocol and experimental conditions applied were as previously reported by González-Centeno et al. [40]. The FRAP reagent was prepared from 0.01 M TPTZ, an aqueous solution of 0.02 M FeCl_3_·6H_2_O and acetate buffer pH 3.6 (1:1:100, *v*/*v*). First, 190 µL of the FRAP reagent was incubated for 10 min at 25 °C. Then, 10 µL of the sample was added, and absorbances were measured at 450 nm every 5 min for 30 min, values of 30 min were used since at this point the reaction was completed.

### 3.7. Colour

Colour determination was performed with a CM-5 colourimeter (Konica Minolta, Tokyo, Japan) with a D65 illuminant and a 2° observer. The colour values were expressed using CIELab* coordinates, where L* represents the luminosity (0 = black; 100 = white), a* the redness (a* > 0) or greenness (a* < 0), and b* the blueness (b* > 0) or yellowness (b* < 0). The figures of psychrometric hue (h*) and chroma (C*) were calculated by equations 1 and 2, respectively. At least 6 values for each cultivar were obtained.

(1) $$h^{*}=a\tan\left(\frac{b^{*}}{a^{*}}\right)$$

(2) $$C^{*}=\sqrt{a^{*2}+b^{*2}}$$

### 3.8. Functional Properties

As functional properties, the fat adsorption capacity (FRC) and the water retention capacity (WRC) were determined. For this, 200 mg of carob pulp powder was weighed in a tube to which 10 ml of vegetable oil (FRC) or distilled water (WRC) were added. The tubes were vortexed and allowed to stand for 24 h. After this time, the tubes were centrifuged for 25 min at 4000 rpm. Then, the liquid supernatant was removed, and the tubes were weighed. The difference in weight is the result of retained fat or water, expressed as FRC and WRC as g/g dm. At least 4 values for each cultivar were obtained.

### 3.9. Statistical Analysis

The variability of the data was analysed with R Statistical Software (R Core Team 4.3.3 version, December 2024), used with RStudio 2025.05.0 [57]. The values are presented with the mean ± standard error. An analysis of variance (ANOVA) and Tukey’s test were carried out to determine differences among cultivars. Correlation analyses between the antioxidant activity and the phenolic and tannin content were evaluated using Pearson’s correlation coefficient. A principal component analysis (PCA) was performed to identify the most significant components and evaluate the contribution of each variable to the separation of the cultivars, focusing exclusively on the significant parameters deemed relevant for the characterisation of carob pulp.

## 4. Conclusions

This study provides valuable insights into the nutritional composition, functional properties, and antioxidant activity of carob pulp powder (CPP) from different cultivars grown in the Balearic Islands. All CPP samples were rich in dietary fibre and sugars—mainly sucrose—highlighting their potential as natural sweeteners and fibre sources in food formulations.

Significant variability among cultivars was observed in protein, sugar, and fatty acid composition, with ‘Des Mestre’ showing higher protein content and cultivars like ‘Orellona’, ‘Santa Maria’, and ‘Roja’ exhibiting higher fructose levels. ‘Roja’ and ‘Orellona’ also had elevated saturated fatty acid content, which could influence flavour and oxidative stability.

Functional properties such as fat and water retention capacity varied across cultivars, suggesting differences in their suitability for specific food applications. Notably, ‘D’en Pau’ demonstrated a high fat adsorption capacity, making it a promising ingredient for fat-binding applications such as in baked goods or meat analogues.

Antioxidant properties, including total phenolic and tannin contents and activity measured via ABTS, CUPRAC, and FRAP assays, also varied. Cultivars like ‘Granja’, ‘Bugadera’, and ‘E-13P’ (‘H2-12’) stood out for their high antioxidant potential, reinforcing their value in health-oriented and functional food products.

Colour differences among cultivars, likely related to flavonoid content, suggest that CPP could serve as a natural colouring agent, offering a reddish-brown hue similar to cocoa.

Principal component analysis highlighted the diversity among carob cultivars. In particular, Ibiza cultivars showed high sugar and stearic acid content but lower antioxidant activity, while open-flower selections were rich in antioxidants and bioactive compounds.

Overall, carob pulp powder offers considerable potential as a multifunctional food ingredient—naturally sweet, fibre-rich, antioxidant, and with functional properties relevant to food formulation and processing. These findings support its application in a wide range of food and nutraceutical products.

## Figures and Tables

**Table 1:** Nutritional parameters of the CPP with no significant (*p* > 0.05) differences among the cultivars.

	Mean	Standard Deviation	Minimum Value	Maximum Value	Confidence Interval 95%
Moisture (g/100 g dm)	14.8	1.7	12.5	19.6	[14.3, 15.4]
Energy value (kcal/100 g dm)	284.3	9.5	263.0	306.0	[280.4, 288.3]
Carbohydrates (g/100 g dm)	60.7	5.5	42.7	70.1	[59.0, 62.5]
Fibre (g/100 g dm)	31.7	5.1	22.6	47.4	[30.1, 33.4]
Sugars (g/100 g dm)	45.9	8.1	22.5	62.5	[43.3, 48.4]
Sucrose (g/100 g dm)	37.6	9.0	14.2	59.9	[34.8, 40.5]
Glucose (g/100 g dm)	4.7	1.6	1.0	9.3	[4.2, 5.2]
Ash (g/100 g dm)	3.1	0.3	2.7	3.7	[3.0, 3.2]
Fats (g/100 g dm)	0.6	0.3	0.1	1.4	[0.5, 0.7]
Butyric acid (g/100 g TFA) ^1^	5.8	3.3	1.8	15.8	[4.8, 6.9]
Linoleic acid (g/100 g TFA) ^1^	13.8	2.4	9.7	20.2	[13.0, 14.5]

^1^ TFA: Total fatty acids.

## Data Availability

Dataset available upon request from the authors.

## Abbreviations

The following abbreviations are used in this manuscript:

AA	Antioxidant activity
ABTS	2,2′-Azino-bis(3-ethylbenzothiazoline-6-sulfonic acid) (radical scavenging assay)
a*	Red/Green coordinate in CIELab* colour space
b*	Yellow/Blue coordinate in CIELab* colour space
CA	Citric Acid
CPP	Carob Pulp Powder
CUPRAC	Cupric Reducing Antioxidant activity (antioxidant assay)
C*	Chroma (saturation) in CIELab* colour space
dm	Dry Matter
Dim1	First principal component of the principal component analysis
Dim2	Second principal component of the principal component analysis
Dim3	Third principal component of the principal component analysis
E410	European food additive code for Carob Bean Gum
EC	European Commission
FRAP	Ferric Reducing Antioxidant Power (antioxidant assay)
FRC	Fat retention capacity
GA	Gallic Acid
GAE	Gallic Acid Equivalents
h*	Hue angle (hue degree) in CIELab* colour space
L*	Lightness coordinate in the CIELab* colour space
PCA	Principal components analysis
SSolids	Soluble solids
TFA	Total Fatty Acids
TC	Total Tannin Content
TE	Trolox Equivalent
TPC	Total Phenolic Content
WRC	Water retention capacity
